# Potential Factors Affected Safety and Efficacy of Transcatheter Plug Closure for Pediatric Hemoptysis with Anomalous Bronchial Arteries

**DOI:** 10.1155/2019/5408618

**Published:** 2019-02-19

**Authors:** Hong-Yu Kuang, Qiang Li, Ping Xiang, Chuan Feng, Qi-Jian Yi, Tie-Wei Lu

**Affiliations:** ^1^Department of Cardiology, Children's Hospital of Chongqing Medical University, Ministry of Education Key Laboratory of Child Development and Disorders, Chongqing 400014, China; ^2^China International Science and Technology Cooperation Base of Child development and Critical Disorders, Chongqing Key Laboratory of Pediatrics, Chongqing 400014, China; ^3^Department of Radiology, Children's Hospital of Chongqing Medical University, Chongqing 400014, China

## Abstract

**Objective:**

To evaluate the safety and efficacy of interventional care in pediatric hemoptysis for anomalous bronchial arteries (BAs) and to identify the potential factors resulting in hemoptysis recurrence.

**Methods:**

20 children complained of hemoptysis were diagnosed with anomalous BAs. All patients received transcatheter plug occlusion in Department of Cardiology, Children's Hospital of Chongqing Medical University. The safety and efficacy were evaluated according to clinical symptoms and images monitoring of enrolled subjects grouped as recurrence group and nonrecurrence group. The potential factors causing hemoptysis recurrence were reviewed and summarized.

**Results:**

No deaths were recorded in a follow-up. Otherwise, hemoptysis recurrence was found in 8 subjects for 14 times, accounting for about 40%. Compared with nonrecurrence group, it indicated a statistical significance in hemoglobin levels (*P*=0.049), mycoplasma pneumonia particle assays (MP-PA) titers (*P*=0.030), and number of anomalous BAs (*P*=0.020). Meanwhile, 50% recurrent scenarios were associated with a respiratory infection by microbiological assessment before transcatheter plug occlusion. The repeat occlusion was applied for unclosed BAs leading to visual recurrent hemoptysis, the average interval time of which was 5.4 ± 3.6 mon.

**Conclusion:**

The data from this retrospective study have shown that transcatheter plug occlusion is a relatively safe procedure with a low mortality. The number of abnormal BAs has been identified as a highly significant predictor of recurrence, and the role of MP and other potential factors should be verified in a multicenter, larger sample size, and randomized controlled trial.

## 1. Introduction

Hemoptysis presents as an expectoration of blood originating from the respiratory system, which can easily lead to a life-threatening condition [1, 2]. A respiratory infection is the commonest etiology in children, while vascular anomaly is a nonignorable contribution in cases of massive blood loss [3, 4]. The diagnostic angiogram has established itself as the* golden criteria* for the diagnosis of an abnormal vascularity, which demonstrated the origins can be traced to the bronchial circulation in 90% cases with respiratory hemorrhage [5]. BAs most commonly originate from the descending aorta at the T5-T6 level, and an aberrant BA was defined as a systemic artery of abnormal anatomy and morphology, along the bronchi. When, beyond the T5-T6 region, it could originate from the aortic arch, the abdominal aorta, the subclavian artery, the thyroid artery, the internal thoracic artery, etc. [6]. Since bronchial arterial embolism (BAE) was identified for the first time in the 1960s, interventional therapy has been considered a safe option [7]. Data from several prior studies have shown recurrence is not uncommon during long-term follow-up after the interventional therapy for hemoptysis that can be caused by various etiologies, such as bronchiectasis, pulmonary tuberculosis, and pulmonary vascular malformations [8]. Currently, although Boudjemline has found that a novel microvascular plug is safe and effective for closure of a variety of vascular anomalies in a short-term follow-up, a prolonged efficacy of transcatheter plug closure for anomalous BAs, a significant burden of blood perfusion, is still a controversy [9]. The aim of this study is to determine the safety and efficacy of transcatheter plug closure and to analyze the underlying potential contributing factor(s) causing recurrent hemoptysis.

## 2. Methods and Materials

### 2.1. Patient Population

This study retrospectively reviewed the clinical data of 20 pediatric patients, who were complained of hemoptysis and received interventional care for the first time in the latest five years (from Nov. 2012 to Oct. 2017). The enrolled subjects comprised 9 males and 11 females aged 9.3 ± 3.0 years of age. All patients were hospitalized and registered in the Department of Cardiology, at the Children's Hospital of Chongqing Medical University (CHCMU). The diagnosis of pediatric hemoptysis was carried out according to a published Chinese standard in 2014. According to clinical records, laboratory examinations, and diagnostic images, the patients were diagnosed with aberrant BAs. Additionally, subjects with the following characteristics were excluded: (1) presence of congenital heart disease (CHD) history; (2) with an imaging confirmation of bronchiectasis; (3) with evident outcomes for tuberculosis infection; (4) highly suspected of idiopathic pulmonary hemosiderosis (IPH) through fiber bronchoscope; (5) did not display an accurate image indicative of bronchial anomalies; (6) had not undertaken an interventional management course of clinical care. This study was approved by the local Ethics Committee of CHCMU. Informed and written consent was obtained from the parents of each patient.

### 2.2. Clinical Assessment and Examinations

According to the estimated standard [10, 11], we created a relative standard of hemoptysis severity based on the volume of blood lost in 24 h per kilogram (20ml-100ml as moderate), in addition to avoiding a bias of weight displayed as follows: <0.71 ml/24 h per kilogram (mild), 0.71-3.56 ml/24 h per kilogram (moderate), > 3.56 ml/24 h per kilogram (massive), > 8 ml/24 h per kilogram which was considered a life-threatening threshold. The preliminary diagnostics included routine blood examination, sputum or gastric juice analyses (sputum culture, sputum for antiresistant Bacillus sp., and iron macrophages), chest X-ray (CXR), computed tomography (CT), CT angiography (CTA), and bronchoscope. For patients who were diagnosed with pneumonia, antibiotics including penicillin, cephalosporin, and macrolides drugs were selected preoperatively. CTA only afforded an approximate view of the vascular anomalies because of an inability of this approach to visually distinguish highly dense tissues and an inability to completely display overlapping tissues, especially for the aberrant BA which is a tortuous route when appearing either congenitally or when acquired [11]. Hence, etiological evaluation indicated a possible diagnosis of vascular anomalies, which consequently indicates a requirement for further digital subtraction angiography (DSA) [12, 13]. Demographic data, clinical characteristics, and images were obtained on first admission of patients to hospital.

### 2.3. Transcatheter Plug Closure

**Figure 1:**
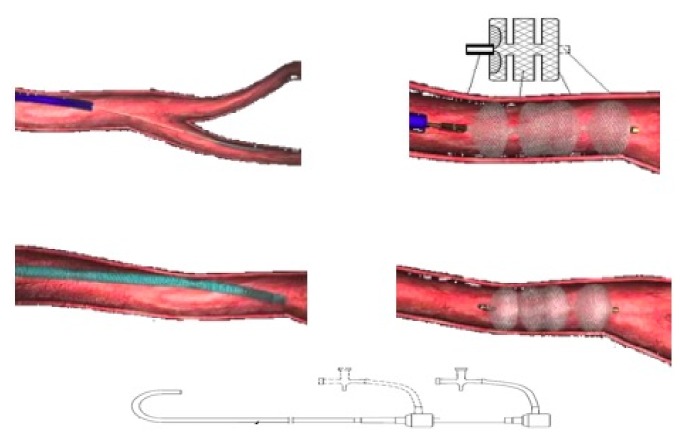
An actual picture of plug device (Starway, Cardi-O-Fix Plug Occluder).

DSA was performed using the transfemoral route using angiographic contrast injected at a dose of 1 ml/kilogram per second from a 5F angiographic catheter (Starway, China). Thoracic aortography was then performed to achieve an approximate view of aberrant BAs or collateral arteries. In addition, selective angiograms identified the anomalous arteries that were characterized by dilation and tortuosity, which clearly paralleled the bronchi and the bleeding sites of target vessels. The vascular plug (Starway Cardi-O-Fix Plug Occluder, Beijing, China) was used in each patient (size of the plug selected was in accord with the diameter of targeted vessels) using a transition system comprising 4F/5F catheters, of which an actual picture was displayed in Figure 1. This procedure was conducted by a professional group that was comprised of two to three cardiologists who had command of the procedure from over 10-year experience, a radiographic professor, and members of the Catheterization Center at CHCMU. Commonly, the plug device was placed on the sites closing to the origin of targeted vessels, and its diameter was 2-4 mm larger than the narrowest one located at the target vessels for loading. In situations where patients had more than one visible abnormal BA with different origins and diameters, we occluded the artery burdening main blood perfusion of vascular lesions. Finally, an instant postocclusion angiograph displayed the volume of residual shunts that was used to evaluate the possibility of postoperative pulmonary hemorrhage.

### 2.4. Follow-Up and Evaluation

Hospital stay (HS, day) was recorded to assess the safety of procedure. After the discharge, the clinical conditions were then tracked by telephone interview, and each patient was commenced with a regular hospital visit for recording of any clinical manifestations, chest radiography, and echocardiogram in the outpatient department of our hospital. When necessary, a repeat DSA could be recommended. A primary endpoint of each hemoptysis scenario has been defined. For every scenario of recurrent hemoptysis, the blood loss, routine blood examination, microbiology, interval time from the last closure, and targeted anomalous vessels were recorded.

### 2.5. Statistical Analysis

Continuous variables were tested for normal distribution and described as mean ± standard deviation (SD) about the mean. For an abnormal distribution, data were displayed as median (interquartile range, IQR), and the nonparametric Mann-Whitney U test was applied. The Fisher exact test was calculated for frequency. An p<0.05 was considered a statistically significant difference. The Cox regression analysis has been applied to identify the predictors of the hemoptysis recurrence, additionally, and furthermore, the cumulative risk of the occlusion was censored by Kaplan-Meier analysis (IBM SPSS Statistics, version 24.0). The analysis of DSA presentation was used to identify the factor(s) that led to recurrence of hemoptysis after occlusion via catheterization.

## 3. Results

### 3.1. Preliminary Outcomes

Among all hemoptysis admissions, three (15%) children presented with a mild hemoptysis, six (30%) children presented with a moderate hemoptysis, and the remaining eleven (55%) children had a massive hemoptysis, among whom 3 patients had a life-threatening condition. Reviewing the history and laboratory analyses of 20 patients revealed that 18 patients (approx. 90%) suffered from a prior respiratory infection with symptomatic hemoptysis, and 75% of WBC counts were more than 10.0*∗*10^9^/L. For microbiological analyses, 15 (77%) patients were found to be associated with increased serum antibody for respiratory MP. For imaging features (i.e., by CXR and CT), among the 20 pediatric patients, we found diagnostic fields in 18 cases (exudative lesions/inflammation/consolidation) and a negative outcome in two cases. On imaging, right-sided lesions were detected in 15 (75%) patients and left-sided diagnostic fields were involved in two (10%) of the patients, and bilateral involvement was found in one (5%) patient. The most frequent zone was centralized to the right inferior lobe. CTA indicated a rough graph of abnormal BAs in 80% of the patients, which predominantly targeted the bronchial vessel (n = 1) originating from the descending aorta at the T4-T6 level (i.e., in approximately 65% of cases). In addition, a BA was detected in the aorta of one case. In two cases, there was evidence of tortuous blood vessels (n≥2). Combined with CTA outcomes, DSA was taken prior to transcatheter plug closure on an individual basis.

### 3.2. Preoperative DSA and Transcatheter Plug Closure

**Figure 2:**
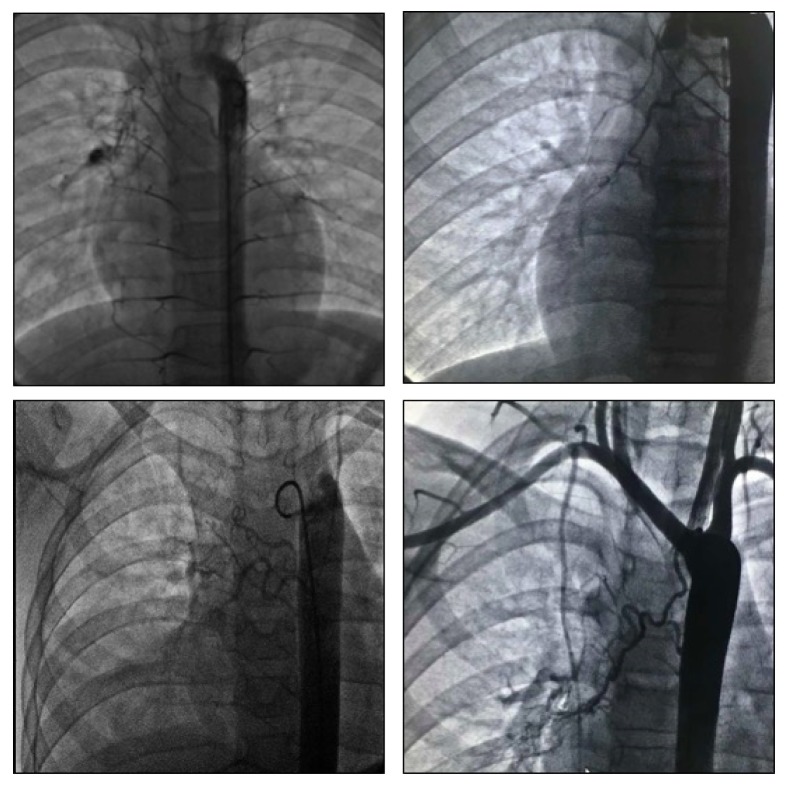
DSA display for anomalous BAs. 2A: the two visible abnormal BAs originated from the thoracic and the abdominal aortas, jointly leading to vascular rupture; 2B: a tortuous BA was clearly presented from the right side of the thoracic trunk, which paralleled the right inferior lobar bronchus; 2C: the right BAs were displayed, and a normal BA paralleled the superior lobar bronchus, while two abnormal BAs with abnormal routes paralleled the inferior lobar bronchus; 2D: two tiny and tortuous arteries apparently repealed, respectively, and originated from the aortic arch and thoracic aorta, forming connecting branches and bleeding in the middle lobe of the right lung.

All cases were confirmed with aberrant BAs by preoperative DSA. In addition, the origin and number of abnormal BAs were identified by aortography. Moreover, the site of vascular rupture was detected by “smoking phenomenon” [12, 13]. In our cases, abnormal BAs were mostly identified from the right wall of the aortic trunk paralleling the right lower lobar bronchus, feeding the parenchyma of the right inferior lobe (Figure 2). The number of aberrant BAs in the first angiography of each subject was recorded and ranged from 1 to 4 with a mean number of 2.05 ± 0.72, respectively, which accounted for 20%, 60%, 15%, and 5%, respectively. Most anomalous arteries were detected from T4 to T7 (n = 27) in 18 cases. We also identified ectopic BAs from the aortic arch (n = 2), the subclavian artery (n = 3), the internal thoracic artery (n = 1), and the renal artery (n = 1). The diameter ranged from 0.3 to 11 mm, with a mean diameter of 1.51 ± 0.50 mm (after removing the extreme data). The selective bronchial aortographic analysis revealed explicit routes for detectable BAs, the communicating branches, and the blood perfusion of bleeding sites in the pulmonary regions. These arteries displayed differential routes and showed rupturing in located lung tissues (Figure 2A, C and D). The first occlusion based on catheterization was carried out for 20 patients, and the vascular plug, of an appropriate diameter relative to the targeted vascular diameter, was loaded using a delivery system. We commonly attempted to occlude the largest aberrant bronchial vessel according to angiographs. However, two cases received an occlusion for two vessels during the procedure. For all patients, once the plug devices were loaded to the targeted vessels, a postoperative angiography showed a significant reduction or disappearance of contrast medium shunts compared to the initial angiography, which was momentarily considered as a successful occlusion. After the procedure, all cases displayed an absence of hemoptysis during hospitalization and patients were discharged with a mean time of 12.7 ± 2.6 days.

### 3.3. Outcomes and Recurrence

At the endpoint, in sum, hemoptysis recurrence was found in 8 patients on 14 occasions, and the interval time from every occlusion was 5.4 ± 3.6 mon. Two patients suffered from a first recurrent hemoptysis within one month, four patients within 6 months, and the remaining two patients no more than 12 months. Three out of eight patients suffered from recurrent hemoptysis on three occasions. The eight patients, including four females and four males, were mainly centralized at school age that ranged from 8.0 to 10.0 years, with a medium age of 9.2 years. The characteristics in the initial hemoptysis scenario were recorded and compared among the recurrence groups and nonrecurrence groups for a detection of potential factors (Table 1). No significant difference was found in terms of gender (*P*=0.535) and age (*P*=0.578). Though patients presented with a less than obvious blood loss, the hemoglobin levels indicated a decreased outcome in children that suffered from a recurrence (*P *= 0.049). At initial, we detected and treated the BAs with obvious anomalies, and yet the neglectful BAs were involved in further recurrent bleeding. Eventually, the number of abnormal BAs in the recurrence group was significantly greater than that found in the nonrecurrence group (*P* = 0.020). It was likely that these arteries that supplied local tissues caused a vascular rupture, which led to subsequent high losses in blood. For each recurrence scenario, the mean volume of blood loss was 102.5 ± 89.7 ml/24 h and ranged from 20 ml/24 h to 350 ml/24h (IQR = 170 ml/24h). Before recurrent hemorrhage, no obvious symptoms in 42.9% of the case scenarios were evident; however, respiratory infectious symptoms were found in eight (about 60%) scenarios within one week and mainly included evidence of cough (28.6%) and fever (28.6%). With regard pathogenic microbiological analyses, MP-PA ≥ 1:320 was detected in 75% patients with a recurrent hemoptysis, which indicated MP acted as an important predictor, possibly.

Eventually, Cox regression analysis has been performed to identify the factors of the recurrence after the procedure for hemoptysis subjects, shown in Table 2. In final multivariate Cox regression model, the number of abnormal BAs (X5) was identified as a highly significant predictor of recurrence, with an adjusted hazard ratio of 3.30 (95% confidence interval 1.14-9.58,* P*=0.028).

**Figure 3:**
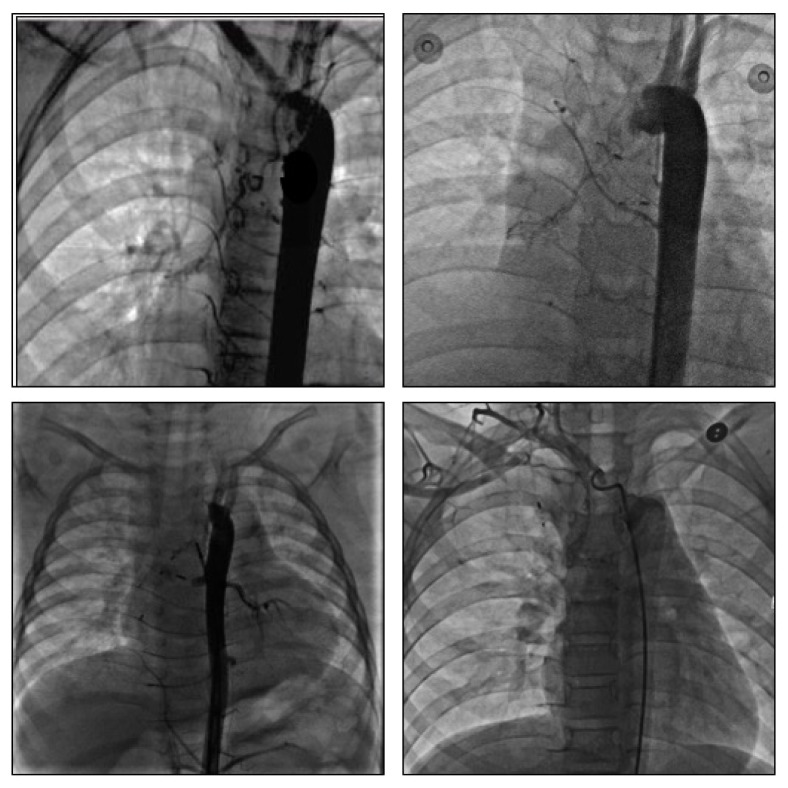
A secondary DSA of recurrent cases that had undergone transcatheter occlusion. 3A: a targeted abnormal BA that originated from the aortic arch was detected, which led to recurrence; 3B: two BAs from the thoracic trunk were blocked off, and a tortuous artery was clearly displayed from the aortic arch; 3C: after transcatheter occlusion of the dilated BAs from the thoracic aorta, another abnormal BA from the abdominal BA was detected in a secondary DSA presentation, which led to a recurrence; 3D: a recurrent case of an abnormal BA was detected with a tortuous route and abnormal origin from the right subclavian artery.

In all recurrent conditions, a repeat angiograph nearly indicated a “smoking phenomenon” in the initial rupture site, while none of the older occluded arteries were found to be involved that led to the reappearance of bleeding. Other unprocessed abnormal arteries initially, associated with some newborn tortuous routes originating from different sites including the descending aorta (above T4-T7), the abdominal aorta, the aortic arch, the subclavian artery, and the thyroid artery, jointly flowed to the segmental pulmonary tissues (Figure 3). These BAs, from the initial angiographs when hemoptysis, were more distant from the lesion, burdening a diffuse contrast with a faint shadow.

**Figure 4:**
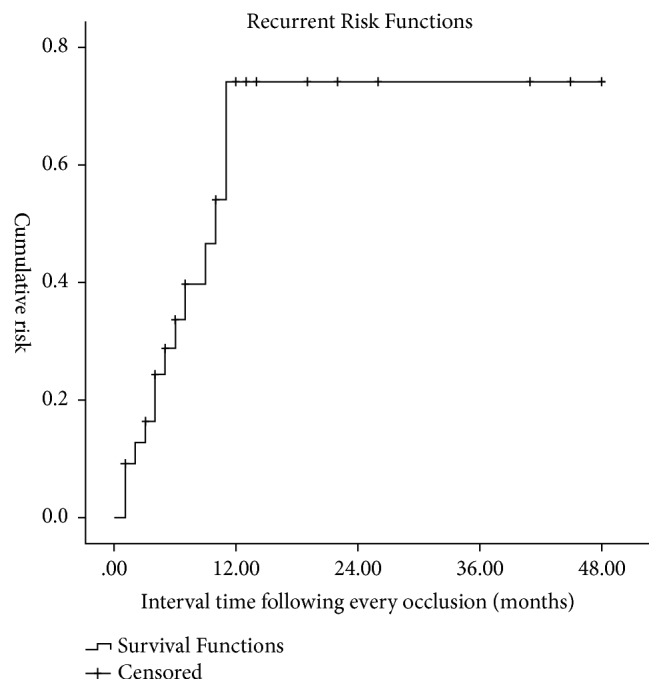
The curve describes the recurrence risk functions for hemoptysis after an occlusion of targeted abnormal BAs, which indicates a high risk following each procedure in the first year.

Therapeutically, repeat plug closure was required. Following the second occlusion, a cessation of hemoptysis was achieved in five patients. In addition, three patients underwent the procedure four times. In sum, 34 occlusions were taken in all patients and it presents as a cumulative risk of recurrence in Figure 4, which indicates a high risk of recurrent hemoptysis in the first year after each successful procedure for occlusion.

### 3.4. Safety

No death and serious adverse events were observed.

## 4. Discussion

Anomalous bronchial circulation plays an important role in pediatric hemoptysis [14]. Since they serve as nutritional vessels, BAs retain a tracking along with the bronchi. Current study demonstrates hemoptysis could be relieved instantly after a transcatheter plug closure, even in a life-threatening hemoptysis condition. Meanwhile, no death and serious adverse events occurred during follow-up, suggesting a safe and efficient procedure. Yet about 40% cases suffered from recurrent hemoptysis and all were considered to be caused by abnormal BAs, during which the blood loss was shown to be significant, this being indicative of a more severe entity. Since there is a significant rate of recurrent hemoptysis, we have analyzed and summarized the underlying factor(s) to provide essential baseline information of the clinical procedures that might possibly refer to the number of anomalous BAs. In the 1980s, there are 10 different anatomic patterns of BAs by angiogram analysis-observations that might underlie recurrent hemoptysis for an incomplete occlusion of all abnormal BAs in a population of 75 hemoptysis patients [15].

We identified eight recurrent cases who had several corresponding arteries with different routes and unequal diameters, originating from the thoracic aorta, the abdominal aorta, the subclavian artery, and the aortic arch. To evaluate the imbalanced distribution of blood flow, the use of conventional angiographic contrast medium can easily reveal the morphology of the main BA trunks, which are responsible for a large share of the blood perfusion. Consequently, a tiny-diameter artery that is inconspicuous can easily be overlooked. In addition, surgeons become accustomed to paying less attention to abnormal arteries of an uncommon origin. This has commonly been demonstrated in the imaging windows in aortic angiography used for observing the anatomy of the aortic arch and the thoracic aorta. Hence, it is important to enlarge the image windows to display every slice, including those of the base of the neck and the upper abdomen, particularly for patients that suffer from bleeding in the lower lobes of the lung, as this bleeding most likely originates from the lower thoracic aorta or the upper ventral aorta. Otherwise, after the bronchial trunks have been occluded, the underlying small arteries and the preexisting tiny BAs will have to accommodate greater blood perfusion to the ruptured vessel [16, 17]. This could increase blood pressure and further enlarge the diameters of other aberrant arteries, which can further damage vascular endothelial cells and enlarge the size of ruptured vessels. Subsequently this can lead to rebleeding and recurrent hemoptysis. Arteries in this area should be systematically searched in order to detect any small BAs that could lead to localized bleeding before catheterization-mediated occlusion [18]. Besides, for the anatomical characteristics of the corresponding branch with a tortuous route and several subbranches, it is more likely to cause a recurrent hemoptysis if they are omitted. One obvious explanation is that tortuous vessels bear a higher blood pressure around at vessel corners. A repeated impact of blood flow can also lead to vascular endothelial damage and eventual vascular rupture. An early occlusion of these tiny and tortuous target vessels is an appropriate selection to prevent recurrent hemoptysis. Mostly, the occlusion of target bronchial and extrabronchial arteries is a major clinical procedure [17, 19, 20]. The abnormal vessels were occluded to cease the symptomatic hemoptysis in the current cases. Though with a similar origination from the systemic circulation, major aortopulmonary collateral arteries (MAPCAs) are considered the primary sources of bleeding in cases of cyanotic CHD that is supplied by restricted pulmonary blood perfusion. Hence in these cases, before CHD surgical management, MAPCAs rarely lead to hemoptysis [21]. These collateral arteries are normally first managed prior to cardiac surgery and are done so in order to decrease blood perfusion from the systemic-pulmonary circulation to avoid reperfusion injury of the lung, to prevent congestive heart failure (CHF) and pulmonary arterial hypertension (PAH), rather than ceasing hemoptysis [21, 22]. The outcomes of our study also demonstrated that recurrence either initially presented with a moderate or massive hemoptysis or with a durable history of hemoptysis presenting as an anemia to different extents and as either a chronic or temporal condition by laboratory tests. Thus, we found that a long-term hemoptysis history and knowledge of the blood volume probably indicated an unsatisfactory prognosis. In the case of a hemoptysis patient that is diagnosed with abnormal BAs and especially associated with a long-term history or an anemic manifestation, it is likely that the interventional occlusion is a good choice at an early stage of discovery. In addition, in each recurrent scenario, it presented as a massive and even life-threatening condition with a blood loss of more than 100.0 ml per 24 hours. It is also possible that there exist multiple sources of bleeding. It is commonly thought that a complete occlusion of every BA could suppress the rate of recurrent hemoptysis in cases that lack restrictive blood perfusion in the pulmonary system. However, it is definitely a life-threatening condition for children with multiple repeats, especially for older pediatric patients. In addition, Gerard et al. have reviewed a series of 60 cases, comparing the recurrent rate, mortality, morbidity, and complications between surgery alone and BAE with lung resection. This group found that BAE as a temporizing measure should probably not be undertaken in patients that are suitable for surgery, since delayed lung resection places these patients at an unnecessary risk of possible fatal recurrent massive hemoptysis [23]. Studies have discussed the possibility that a lobectomy or a bilateral lung resection may lead to an improvement in the quality of life, especially for patients with hemoptysis caused by mixed pulmonary arteries of unclear origin [24]. Additionally, all twenty cases studied in this report were primarily admitted to hospital when accompanied with a respiratory infection, which has been identified as a common etiology for an enlargement of BAs [25]. Bronchial arterial inflammation and endothelial cell lesions could secondarily lead to the acquired BA abnormality of vascular wall weakening, which probably acted as an inducing factor of the rupture of target vessels, causing hemoptysis since it is capable of eliciting a dilated vessel, thereby leading to a gradual increase in blood flow to the pulmonary tissues and a vascular rupture [13, 26]. A chronic inflammatory reaction may postpone the expected time of alleviation in hemoptysis patients with a lower rate of respiratory infection. The inflammatory response that occurs in the pulmonary tissues can induce dysfunction of blood-oxygen transfer [27]. Patients with an infection could present with dysfunctional pulmonary ventilation, which essentially occurs through the respiratory membrane. Intravascular endothelial cells, as important components of the respiratory membrane, are commonly destroyed once the lung is infected by diverse pathogens, especially in the context of an MP infection [28]. These bleeding sites provide the optimal environment for other bacteria to cause an additional super-infection of the respiratory system and to possibly exacerbate the damage to vascular endothelial cells, thus eventually leading to a pernicious cycle of events. In addition, mass secretions, such as sputum or bloody discharges, are built up in the respiratory system of patients with a lasting pulmonary infection. Even if closure of the aberrant vessels has been attempted, a lasting infection in pulmonary tissues can gradually invade the capillaries, eventually causing the destruction of the lung parenchyma and the erosion of resident local blood vessels. Investigations have shown that many factors, such as emotion/stress, trauma, pressure, and an inflammatory reaction, can stimulate local bleeding in circuitous vascular sites. The affected lung tissues are partly supplied by tiny bronchial vessels, and a sharp increase in pressure may increase the internal diameter and the blood flow rate in these tiny vessels, again causing rupture of the vascular terminals of previous communication branches or partial blood capillaries, leading to formation of a fistula that is seen on imaging and causing recurrent hemoptysis. Standard medical management for infections should be followed after the interventional therapy to control the inflammatory response, to minimize the damage to endothelial cells and the respiratory membrane.

Hence, the number of abnormal BAs, anemia manifestation, hemoptysis severity evaluation (i.e., volume of blood loss), respiratory tract infections, etc. are likely the risk factors for recurrent hemoptysis following transcatheter occlusion. Moreover, a late childhood group probably suffers more from this condition according to our data. For the above reasons, recurrent hemoptysis is not rare, and yet it presents as a potential life-threatening condition in children. Eventually, a Cox regression analysis showed that the number of abnormal BAs was an independent prognostic factor for hemoptysis recurrence after an occlusion (*P*=0.028). So, for targeted vessels, a relatively complete occlusion, not only of the main trunk, but also of the visually tortuous and smaller vessels, is employed to lower the risk of recurrent hemoptysis. In a severe condition with above factors, a segmental lung resection should be taken into consideration to decrease the risk of rebleeding episodes [29, 30].

## 5. Conclusions

The transcatheter plug closure is a safe and efficient procedure for hemoptysis caused by anomalous BAs, however, which could be influenced by many potential factors. Based on the outcomes of this single-center study with a relatively small sample size, our study calls for a multicenter, larger sample size, and randomized controlled trial to verify our observations reported herein.

## Figures and Tables

**Table 1:** Characteristics of enrolled cases between groups.

Characteristics	All	Recurrence^*∗*^	Non-recurrence	*P* value
N	20	8	12	-
Sex(male)	9	4	5	0.535
Age (year)	9.3(3.0)	9.2(1.6)	9.3(5.7)	0.578
Blood loss (ml/24hr per kg)	4.46(4.32)	6.26(3.95)	3.46(3.64)	0.203
RBC (10^12/L)	4.11(0.88)	4.0(1.10)	4.24(0.65)	0.355
Hb^†^ (g/L)	116.5(29.5)	104.0(24.0)	124.5(19.5)	0.049^¶^
WBC (10^9/L)	8.53(4.54)	8.53(3.90)	8.71(5.29)	0.817
NEUT (%)	69.5(24.5)	72.0(28.5)	69.5(23.5)	0.877
MP-PA (+) ≥1:320 titers(n)	10	6	4	0.03^¶^
Abnormal BAs (Mean ± SD) ^**∗***∗*^	2.35 ± 1.07	3.13 ± 1.27	2.0 ± 0.58	0.02^¶^
HS (day)	12.7(2.6)	12.5(4.0)	12.5(4.0)	0.459

Value displayed as medium (IQR) except for the abnormal BAs counts.

^**∗**^The cases in the recurrence group were readmitted to hospital for recurring hemoptysis during follow-up.

^†^Hb=hemoglobin. ^**∗***∗*^The number of abnormal BAs in angiographs. ^¶^A statistically significant difference.

**Table:** (a) Variables in Cox regression analysis

Column	Variables	Explanation
Age	X1	Year
Gender	X2	Male=1; Female=2
Hemoglobin	X3	<110 =0; ≥110=1
MP-PA (+) ≥1:320 titers	X4	<1:320=0; ≥1:320=1
Abnormal BAs*∗*	X5	Number
Time	t	Month
Status	Y	Recurrence=1

*∗* indicates the number of abnormal BAs in angiographs; MP-PA=mycoplasma pneumonia-particle assays.

**Table:** (b) Variables in the equation

	B	SE	Wald	df	Sig.	Exp(B)	95.0% CI	
							lower limit	upper limit
X1	-.133	.249	.284	1	.594	.876	.537	1.428
X2	1.532	1.030	2.211	1	.137	4.626	.614	34.832
X3	.260	.988	.069	1	.793	1.296	.187	8.989
X4	1.410	1.126	1.568	1	.211	4.094	.451	37.197
X5	1.194	.544	4.825	1	.028	3.301	1.137	9.582

## Data Availability

The data used to support the findings of this study are available from the corresponding author upon request.
